# Supplementary material for: Policymaking through a knowledge lens: Using the embodied-enacted-inscribed knowledge framework to illuminate the transfer of knowledge in a mental health policy consultation process – A South African case study
Source: PLoS One. 2021 Jan 13;16(1):e0244940. doi: 10.1371/journal.pone.0244940 (PMC7806173; doi:10.1371/journal.pone.0244940)
Supplement: S1 Table — (DOCX) [file pone.0244940.s001.docx]

**S1 Table: Types of knowledge claims coding framework**

| **Code** | **Coding rule / definition** |
| --- | --- |
| **Evidence-based knowledge** | All references to studies, research, data, literature/theories or international ‘best practice’ guidelines, regarding what is shown to be effective (e.g. interventions) or problematic (e.g. risks, treatment gap etc.) |
| **Experiential knowledge** | All references to first or third person accounts or observations of on-the-ground experiences of problematic issues/challenges or effective/ineffective approaches in practice |
| **Other** | All other references in talk that do not fit into either of the two coding rules above (e.g. clarifications, introductions, procedural comments etc.) |
