# Supplementary material for: Policymaking through a knowledge lens: Using the embodied-enacted-inscribed knowledge framework to illuminate the transfer of knowledge in a mental health policy consultation process – A South African case study
Source: PLoS One. 2021 Jan 13;16(1):e0244940. doi: 10.1371/journal.pone.0244940 (PMC7806173; doi:10.1371/journal.pone.0244940)
Supplement: S2 Table — (DOCX) [file pone.0244940.s002.docx]

**S2 Table. What knowledge claims were being used to do coding framework**

| **Main theme** | **Sub-theme** | **Coding rule / definition** |
| --- | --- | --- |
| **Illustrate current situation** | Illustrate a challenge | All knowledge claims making reference to a current “on-the-ground” situation, whether experienced in first or third person, or to evidence, to illustrate a challenge that needed addressing |
|  | Illustrate a solution / best practice | All knowledge claims making reference to a current “on-the-ground” situation, whether experienced in first or third person, or to evidence, to illustrate a solution or demonstrate a best practice that was currently working |
| **Highlight implications of a proposal** | Highlight benefits of or motivate for a proposal | All knowledge claims making reference to a first or third person experience, or to evidence, in order to explicitly link to and demonstrate how a proposal being discussed would work or be of benefit in this situation |
|  | Highlight disadvantages or argue against a proposal | All knowledge claims making reference to a first or third person experience, or to evidence, in order to explicitly link to and demonstrate how a proposal being discussed would not work or be disadvantageous in this situation |
| **Engage** | Support a previous point made | All knowledge claims – whether in reference to experience or to evidence – that were explicitly aimed at engaging with and supporting or building on a previous point made |
|  | Counter a previous point made | All knowledge claims – whether in reference to experience or to evidence – that were explicitly aimed at countering a previous point made |
