# Supplementary material for: Policymaking through a knowledge lens: Using the embodied-enacted-inscribed knowledge framework to illuminate the transfer of knowledge in a mental health policy consultation process – A South African case study
Source: PLoS One. 2021 Jan 13;16(1):e0244940. doi: 10.1371/journal.pone.0244940 (PMC7806173; doi:10.1371/journal.pone.0244940)
Supplement: S3 Table — (DOCX) [file pone.0244940.s003.docx]

**S3 Table. Responses to knowledge claims coding framework**

| **Code** | **Coding rule / definition** |
| --- | --- |
| **Responded to (overall)** | Any knowledge claim made during the group discussions that was verbally responded to in some way, acknowledging the point made (code further divided below) |
| **Responded to and engaged with** | All knowledge claims to which there was a verbal response and follow on or engagement with the point made within that knowledge claim (such as reframing, summarising, building on, countering) |
| **Responded to but not engaged with** | All knowledge claims to which there was a verbal response of acknowledgement but with which there was no further engagement in terms of following through on content (e.g. thank you, yes) |
| **Not responded to** | All knowledge claims that were made during discussions that received no verbal response from group Chair or other group participants (discussion moved onto other points or participants) |
| **Inaudible** | All knowledge claims within discussions that could be identified but for which the subsequent talk was not sufficiently audible to determine type of response / non-response |
