# Supplementary material for: Policymaking through a knowledge lens: Using the embodied-enacted-inscribed knowledge framework to illuminate the transfer of knowledge in a mental health policy consultation process – A South African case study
Source: PLoS One. 2021 Jan 13;16(1):e0244940. doi: 10.1371/journal.pone.0244940 (PMC7806173; doi:10.1371/journal.pone.0244940)
Supplement: S4 Table — (DOCX) [file pone.0244940.s004.docx]

**S4 Table. Reflection / inscription of knowledge claims in group recommendations coding framework**

| **Code** | **Coding rule / definition** |
| --- | --- |
| **Reflected or inscribed** | Group recommendation reflects a comprehensive direct or summarised version of knowledge claim made |
| **Partially reflected or inscribed** | Group recommendation partially reflects the content of a knowledge claim, with some detail lost in the inscription |
| **Not reflected or inscribed** | Group recommendation does not reflect the content of knowledge claim in any way |
