# Supplementary material for: Policymaking through a knowledge lens: Using the embodied-enacted-inscribed knowledge framework to illuminate the transfer of knowledge in a mental health policy consultation process – A South African case study
Source: PLoS One. 2021 Jan 13;16(1):e0244940. doi: 10.1371/journal.pone.0244940 (PMC7806173; doi:10.1371/journal.pone.0244940)
Supplement: S5 Table — (DOCX) [file pone.0244940.s005.docx]

**S5 Table. Examples of knowledge claim types**

| **Knowledge claim type** | **Examples** |
| --- | --- |
| Evidence-based knowledge claims | The second point related to that is the discussion about task shifting because we all within our different professional categories have historically performed certain roles. But a lot of the discussion in World Health Organization and in mental health literature in recent years has been around the issue of task shifting. So, for example, in the UK, there’s a specific programme to train nurses in specific aspects of cognitive behavioural therapy for depression and other common conditions. Or in India, there are programmes to train community health workers in interpersonal group therapy for depression. And I think that question of task shifting and what is done at what level is quite critical. (Speaker 9, Group 4)  I was reading this book called *The silent cure* by [Helen Epstein], not sure if folks have read it, but it's really an account – she's a medical anthropologist and epidemiologist that has done a lot of work on the continent. And she calls attention to the fact that the average number of sexual partners among people in Africa is really no different from the average number of sexual partners in North America and Europe. It’s the same ballpark figure. So, it’s not clear that it’s the number of sexual partners that’s the issue, but what she does call attention to is the different kinds of sexual networks that operate on this continent compared to other continents. (Speaker 3, Group 5)  And also what we don’t know is, we don’t know what happens in the rural areas. Because if you look - if you’ll allow me just to mention this book of mine, which is suicidal behaviour in South Africa, which gives quite a lot of data, but, if you look at the studies that I quote in here, most of them are based on hospital and mortuary statistics. We don’t really know what happens in the smaller towns and villages where there is a big problem. (Speaker 3, Group 9) |
| Experiential knowledge claims | I just want to share … in correctional services, because of the nature of the thing, of the correctional environment we tried to ‘verticalise’. You know what happened? HIV services, mental health services died. Because this nurse says, I’m only doing chronic disease, I won’t do mental health, I won’t do HIV and if that nurse is not there for a month, it means, patients won’t get their treatment. I think for three to four years, we trying to reverse that but the nurses say, you said we must do this only and we won’t do the others. (Speaker 26, Group 1)  I still miss the days when our psychiatric community sisters had cars. And they could drive to the patient’s home, and they would come to Chris Hani Baragwanath hospital with their files and they would tell us exactly what’s going on and they’d say “You know what? So-and-so didn’t come for an appointment. I’m just going to pop down and see what’s going on at the house”. What’s happened to that? Talk about community care. Let me understand what’s happened to the community psychiatric nurse in her car, who lived and understood and knew the community that she serviced. And would liaise with us and say “Hey, watch out for so-and-so because we haven’t seen them for a while. Don’t know what the hell’s going on. Be on the lookout”. What’s happened to that? Where’ve they gone? I don’t know. It’s very sad. Funding? Yes. Funding. Organisation. Administrative efficiency. (Speaker 1, Group 3)  What I’ve done this morning is bring you – I would like to just pass them round – some copies of what has been in the paper recently. I’ll tell you what my concern is. I’m on the board of an NGO, and the health department has suddenly, for no reason, stopped paying them. They have done that to a lot of NGO’s, this particular one that I’m passing around is the [name of NGO]. Now, [NGO name] went to the paper… Now, these are the people that are not being paid. And I think this is just so horrendous and so important that I think it should take precedence very much because when it gets to the situation that we have to rectify this situation through going to the media, I think it is chronic. (Speaker 32, Group 10) |
