# Supplementary material for: Policymaking through a knowledge lens: Using the embodied-enacted-inscribed knowledge framework to illuminate the transfer of knowledge in a mental health policy consultation process – A South African case study
Source: PLoS One. 2021 Jan 13;16(1):e0244940. doi: 10.1371/journal.pone.0244940 (PMC7806173; doi:10.1371/journal.pone.0244940)
Supplement: S6 Table — (DOCX) [file pone.0244940.s006.docx]

**S6 Table: Examples of functions of knowledge claims**

| **Theme** | **Sub-themes** | **Knowledge claim type and examples** |
| --- | --- | --- |
| **Illustrate current situation** | Challenge | **Evidence-based** |
|  |  | In fact, in another study, which I haven’t spoken about here, we are conducting interviews with patients who meet the diagnostic criteria for major depressive disorder, and we are trying to elicit from them, in the local expressed language – Xhosa in this case – the manifestations of depression from their own perspective … And what we are finding is that a lot of people are talking about the somatic dimension of depression. People are reporting pain and idioms of distress, such as “My heart is sore”, “My mind is not right”, those kinds of idiomatic expressions of distress. And finding the language to be able to express that is part of the challenge that we are trying to engage with. (Speaker 3, Group 5) |
|  |  | **Experiential** |
|  |  | Then my other concern is, as a community member of the mental health review board, we’re supposed to take this information and all that to the community, be a resource in the community. But then many a times we find that that the patient says, the user says, that “The medication I’m getting is not treating me well; there’s a lot of side effects. And if I go to work, I’ve got this mask face, and immediately people recognise that and, you know, that’s where the whole stigma and discrimination starts all over again”. So, many a time, the psychologists, the doctors, doesn’t wanna listen to us, the user, or even, you know, as a board member that try and adjust the medication so that this person can function as normally as possible in the community. Those other are the nitty gritty’s that I thought that would be discussed here. (Speaker 34, Group 10) |
|  | Solution/best practice | **Evidence-based** |
|  |  | And then fourthly I wanted to mention the WHO norms for mental health, which includes norms for human resources. I don’t know if people are familiar with them but in fact the lead author on that was (name) … who spoke yesterday. And that model, which is available online, follows a fairly similar process to the one that (presenter) has outlined and from that one’s able to look at looking at staff norms based not just on a needs-based or epidemiological approach, but on a demand-based approach, which I agree is really the way to go. (Speaker 9, Group 4) |
|  |  | **Experiential** |
|  |  | If I may share some of what we’re finding, is working on the ground as an NGO … We are trying to roll out psycho-social services for children in a children's home with a zero budget and only one qualified psychologist. And we’re having quite a lot of success in terms of task shifting. We are using volunteers from all over. A lot of pupilships; large companies are sending us volunteers so that we can then deploy them playing soccer with the children, helping them with homework … We're trying to see how we can, with zero money – or as little as possible – bring the services to the kids who really need them, and we're finding that using volunteers and interns is a wonderful pathway. (Speaker 11, Group 7) |
|  | | |
| Table S6 cont.: Examples of functions of knowledge claims | | |
| **Theme** | **Sub-themes** | **Knowledge claim type and examples** |
| **Highlight implications of a proposal** | Highlight benefits of, or motivate for, a proposal | **Evidence-based** |
|  |  | Can I also say, and that the evidence shows, that we know like the one you need a bit more than one counselling on site. Yes, have it on site, initially, but I mean what the international evidence shows that the most effective programmes, and here we’re talking about mental health promotion for maternal depression and promoting psycho-social stimulation which can have long term impact, twenty years later ((inaudible)) is through home visitation programmes. Because you know we can’t be guaranteed that mother who’s got maternal depression’s going to come back to the facility. (Speaker 1, Group 1) |
|  |  | **Experiential** |
|  |  | I started reading *On recovery* about a year ago, and personally, I can say that there’s been a fundamental change in my own levels of job satisfaction and enjoyment. The reward when you start seeing the positive, and helping people to see that and bring it out is just immense … The point about the recovery movement is that we create a force that actually allows us to get the job done – not only get the job done, but improve society as well. (Speaker 3, Group 10) |
|  | Highlight disadvantages of, or argue against, a proposal | **Evidence-based** |
|  |  | Please, can I just say, I think that it’s a bit of a problem that we can put wonderful things here, but what is actually possible and feasible within our resources of today? So, I would endorse that we need to promote strong families or whatever, but, if we’re looking at programmatic interventions to strengthen families, both (name) and I have been involved in a random controlled trial of a family strengthening programme, and it’s quite resource intensive. You know, so you need warm bodies to actually facilitate those programmes … So, the issue is, do we use our limited resources for everybody? That’s really nice, but in a resource-constrained situation, which we have, do we try and rather target those particularly in need of a strengthening programme? (Speaker 1, Group 1) |
|  |  | **Experiential** |
|  |  | I’d like to say something coming from the ground. I’d like to comment on the issue of dedicated person at each facility. Being a person who is working on the ground, I have observed that really if you have a dedicated person in a clinic who is doing mental health, you tend to have a relationship with your patients. The patients, when they come they know who to contact and whenever they start to develop either side effects or whatsoever, they are very open. And when they change or when introduction of integration started, whereby any professional has started could render mental health services, we realised that it led to default rate, because patients were not having relationships with a particular nurse and some of the nurses were not even having passion in mental health. (Speaker 28, Group 3) |
|  | | |
| Table S6 cont.: Examples of functions of knowledge claims | | |
| **Theme** | **Sub-themes** | **Knowledge claim type and examples** |
| **Engage** | Support previous point | **Evidence-based** |
|  |  | I agree with (name). I think under-reporting is a big problem in this country, mainly because of the distribution of people. Rural versus urban. The second point that I just want to support (name), is that the study that I just completed in the Durban, the three Durban mortuaries, showed me conclusive evidence that the reporting leaves a lot to be desired in the mortuaries. (Speaker 4, Group 9) |
|  |  | **Experiential** |
|  |  | I’m from a district hospital in Kwazulu-Natal, and this slide here pleases me so much. This is exactly what is happening on the ground, in our district. Only one thing I’d like to add to the clinic, the need for a professional mental health person and a facility for the clinic to communicate with and give the necessary intellectual input, pharmacological input, care input where necessary, is absolutely essential. So from that clinic’s picture there, I’d like to have an arrow to the district hospital. In our district hospital, we’re very lucky; we’ve established a psychiatric unit, so we’re able to care for members of the community that require the expertise of a psychiatric unit and give feedback and support to people at the clinics. (Speaker 18, Group 3) |
|  | Counter previous point | **Evidence-based** |
|  |  | I want to slightly disagree with (name). I think we need to be more vocal. We need to be advocates for the mental health sector and I think the national Minister for Health is very serious about the concern. Being the third highest burden of disease, we need to plough in a hell of a lot more when it comes to funding. So, I want to suggest that we call for ring-fencing, with clear indicators as to what it is we want to achieve by when. You know, there’s already a massive gap in the finding stream. We heard yesterday about the study that was done in Kwazulu-Natal - psychiatric services received over a five-year period about 3.5 percent increase, whereas general health received 10, between 10.5 and 12 percent increases. Already we see that disparity. If we want to reduce the impact of this burden, reduce the economic burden, then we’ve gotta plough more money into ensuring that we lower the burden. (Speaker 46, Group 3) |
|  |  | **Experiential** |
|  |  | What I would like to say is that I do understand what my colleague is saying, that they would like to expedite the matter and things, but in terms of the act if you look at it, the reason we actually give two weeks from the date of when you receive the appeal … is to allow the applicant to request legal representation from the legal aid if they so wish. And another valid point is also that, as the review board, we have to issue summons for the psychiatrists and health care practitioners to make themselves available to come and give evidence in the hearing. (Speaker 23, Group 6) |
