# Supplementary material for: Policymaking through a knowledge lens: Using the embodied-enacted-inscribed knowledge framework to illuminate the transfer of knowledge in a mental health policy consultation process – A South African case study
Source: PLoS One. 2021 Jan 13;16(1):e0244940. doi: 10.1371/journal.pone.0244940 (PMC7806173; doi:10.1371/journal.pone.0244940)
Supplement: S7 Table — (DOCX) [file pone.0244940.s007.docx]

**S7 Table: Examples of responses to knowledge claims**

| **Response type** | **Examples of responses** |
| --- | --- |
| Responded to but not engaged with | **Evidence-based** |
|  | Thank you for that. (Speaker 2, Group 5)  And this is the place to do it. Question there, okay this will be the last question and then we can save the others for later. (Speaker 2, Group 5) |
|  | **Experiential** |
|  | Okay, so we’ve got that one solved. We’re packing it away now. There’s two other issues I want you to address before you can go to tea in two minutes’ time. (Speaker 1, Group 3) |
| Responded to and engaged with | **Evidence-based** |
|  | That’s a good point you’re making. I mean, with all that you’ve said there, you’ve made the point that why so few are children involved in sports at school. I will give you lots of reasons. Schools don’t have facilities, schools have attitudinal issues, all sorts of issues. (Speaker 9, Group 1) |
|  | **Experiential** |
|  | Okay. What you saying there is very important. You are saying people with skills in psychiatry and understanding are not involved in the infrastructure planning. Okay, that’s vital, and that should happen. That expertise should come there. (Speaker 3, Group 6) |
