# Supplementary material for: Policymaking through a knowledge lens: Using the embodied-enacted-inscribed knowledge framework to illuminate the transfer of knowledge in a mental health policy consultation process – A South African case study
Source: PLoS One. 2021 Jan 13;16(1):e0244940. doi: 10.1371/journal.pone.0244940 (PMC7806173; doi:10.1371/journal.pone.0244940)
Supplement: Attachment — Submitted filename: Response to Reviewers.doc [file pone.0244940.s008.doc]

Dr Debbie Marais

Faculty of Medicine and Health Sciences

Stellenbosch University

South Africa

Dr Joseph Telfair

Academic Editor

*PLOS ONE*

12 December 2020

Dear Dr Telfair,

Thank you for your feedback and the reviewer’s comments on our manuscript, “*Policymaking through a knowledge lens: Using the embodied-enacted-inscribed knowledge framework to illuminate the transfer of knowledge in a mental health policy consultation process – a South African case study”.* We appreciate the feedback and believe that the reviewer’s input has assisted us to improve this paper. Please find our responses to these comments in the table below, with reference to the pages in the manuscript where these changes have been made, in track changes.

Thank you again for your consideration of this manuscript.

Sincerely,

Debbie Marais

**Responses to r**eviewers’ comments

| **Academic Editors’ Comments** | | | |
| --- | --- | --- | --- |
| **#** | **Comment** | **Response** | **Manuscript reference** |
| 1 | Please ensure that your manuscript meets PLOS ONE's style requirements, including those for file naming. | The style guide and file naming requirements have been carefully attended to and the manuscript and corresponding files amended accordingly. | Manuscript and supporting files |
| 2 | We note that you have indicated that data from this study are available upon request.   a) If there are ethical or legal restrictions on sharing a de-identified data set, please explain them in detail (e.g., data contain potentially sensitive information, data are owned by a third-party organization, etc.) and who has imposed them (e.g., an ethics committee). Please also provide contact information for a data access committee, ethics committee, or other institutional body to which data requests may be sent. b) If there are no restrictions, please upload the minimal anonymized data set necessary to replicate your study findings as either Supporting Information files or to a stable, public repository and provide us with the relevant URLs, DOIs, or accession numbers. | Data cannot be publicly shared because there are ethical restrictions on sharing the complete data set. The data comprises verbatim transcripts of discussions in each of the ten breakaway groups, with a particular focus on Chairs’ input. Although permission was obtained by the Department of Health to record these sessions, this was not explicitly provided to use the information for research purpose. The group of participants participating in these discussions, and the Chairs of these sessions, would be potentially identifiable from their narratives and the way they spoke about their experience and positions as part of their input to the policy discussions, despite the use of participant codes to de-identify the data. Sharing the full data set would violate the principle of confidentiality.  For researchers who meet the criteria for access to confidential data, the Biomedical Research Ethics Committee at the University of KwaZulu-Natal (South Africa) can be contacted to request permission to access the data (contact: Ms Anusha Marimuthu, +27312604769, [BREC@ukzn.ac.za](mailto:BREC@ukzn.ac.za)). | N/A |
| 3 | Please include captions for your Supporting Information files at the end of your manuscript, and update any in-text citations to match accordingly. | Captions for Supporting Information files have been added at the end of the manuscript. In-text labels and file names have been updated according to the guidelines. | Supporting information captions: pp. 30-31  In-text labels: pp. 11,13,15  Supporting files |
| **Comments from Reviewer 1** | | | |
| **#** | **Comment** | **Response** | **Manuscript reference** |
| 1 | Introduction: The introduction was well written but one element that could be improved is to have a single and clear aims statement. Currently, the authors state their aims at the bottom of page 5 (lines 116-118) but also at the beginning of the section “Context of the study” (lines 163-164). I propose that the authors reformulate their sentence at the beginning of the context section to ensure there is no confusion as to the main aims of the study. | The aim in the Context section has been rephrased to align with the earlier stated aim. | Context: p. 5 |
| **#** | **Comment** | **Response** | **Manuscript reference** |
| 2 | Methods: The authors provide adequate description of their data sources and analysis process but there is very little information provided about the participants of the summit and each of the breakout sessions. We learn only in the discussion that there appeared to be few service users in attendance but the composition of participants in the breakout sessions should be reported, as it will influence the types of knowledge claims made during these sessions. For instance, were there service users present in every breakout session or only some sessions? How many? | A full list of summit participants was not available (or perhaps even recorded). A foreword of the final policy document states that the summits were attended by representatives from “research groups, academia, professional associations and statutory health institutions, the World Health Organization, nongovernmental organizations, mental health care user groups, clinicians, national and provincial departments that play a role in mental health.”.  Similarly, a list of participants at each breakaway group was either not recorded or not made available by the Department of Health. Summit participants self-selected into different breakaway groups. It was possible to identify many of the speakers from the audio-recordings as they were either identified by the Chair or self-identified. Most of these were either academics or service providers (or both), although more service users were identified in Group 10 (on Advocacy, social mobilisation, user and community participation).  These contextual issues (including participant details) have been described in an earlier paper.  In addition, the lack of representation of service users at the summit was identified as an issue by representatives from service user groups who were interviewed during an earlier phase of this study. These findings are reported on in the earlier paper.  A reference to a more detailed description of the context and participants in the summit being provided in an earlier paper has been included in the Methods section of this paper. | Methods: p. 10 |
| 3 | Methods:  Similarly, in the description of the national summit and breakout sessions some details could be provided in the context section on the chairs/facilitators of the breakout sessions and the context within which the discussions took place (e.g. time constraints). | These procedural issues (including facilitation by Chairs) were reported on in the previous paper referenced above.  Reference to the description of procedural issues in the previous paper has been included in the Methods section of this paper. | Methods: p. 10 |
| **#** | **Comment** | **Response** | **Manuscript reference** |
| 4 | Methods:  One element missing from the description of the analysis is which research team members participated in the analysis and how this was done. Was the analysis carried out by a single person or a team, with what experience or background, etc. | The principal author, a mental health professional who had experience in qualitative analysis of complex data sets in multi-country projects, conducted the analysis. Regular meetings were held with co-authors to discuss and verify both the analysis process and the application of themes (e.g., knowledge form codes) across the data set. This detail has been included in the Methods (data analysis) section. | Methods – data analysis: p. 10 |
| 5 | Methods:  Small detail, but given how the results are reported, it would seem sensible when presenting the four lines of analysis (page 10, line 214) to re-order the information so that the functions of knowledge claims appears before the responses to knowledge claims. | Thank you for this useful feedback. The “responses to” and “functions of” categories have been swapped around in the Methods section to correspond with the Results section. | Methods: pp. 10-11 |
| 6 | Results/Discussion:  One of the authors’ main findings is that participants’ knowledge claims (whether evidence-based or experiential) were largely not reflected in the group recommendations made during the summit’s plenary session. The authors suggest that this indicates that participants’ knowledge claims may not be sufficiently captured and inscribed during the consultation process, which in theory runs contrary to the purpose of the consultation process itself. However, one thing that was less clear was whether the national summit was held principally as a way to validate and ensure acceptability of policy directions determined by the government or whether the goal really was further development of these policies. If the goal was the former, it would explain why only a small number of knowledge claims were considered during the plenary discussions, as the goal may have been to engage in a consultation exercise without actually challenging the policies too much. In other words, was the government’s approach more political than instrumental? Were the goals of the national summit explicitly stated in any documentation surrounding the event? | It is stated in the forewords in the final policy itself that the purpose of the provincial and national summits was to develop and finalise the draft policy. A previous paper described interviews with participants at both the provincial and national summits, describing how the same draft policy was available at the national summit as had been at the provincial summit. This paper also reports that a draft of the ‘summit resolution’ from the national summit was already available to participants before discussions began. Some interviewees had been convenors of the provincial and national summit themselves and reported having no clear direction regarding how inputs should be captured or used, as well as a sense that the consultation process was a rubberstamping exercise – a purpose that was not made clear to the over 4000 participants in the provincial and national summits, some of whom were interviewees who reported feeling misled. If the purpose of the summits was to endorse the policy, there was still no systematic process for capturing participants’ inputs from across the breakaway group discissions in order to check this against the existing policy and confirm this, which was particularly important given that no government officials mandated with the policy were not in attendance at these discussions. These findings are reported on in this previous paper.  A reference to the lack of clear purpose described in the previous paper has been added to the Context section of this paper. | Context: p. 9 |
| **#** | **Comment** | **Response** | **Manuscript reference** |
| 7 | Results/Discussion:  Another related issue is that since the paper’s focus is on the micro events during the national summit, we have little information on how the earlier provincial summits informed the draft policy that was presented during the national summit or to what extent the final policy plan (post summits) may have been informed by different forms of knowledge during the development process. It would be reasonable to think that there may have been a more complete capture and transformation of knowledge during the initial rounds of consultations (when the policies really were being developed) than during the national summit when the government had already determined its policy directions and the justification for them. The paper would have us conclude that the government may not have used appropriate methods to capture the views of different stakeholders, but can that conclusion really be made without considering the information and knowledge shared by participants during those provincial summits? | The similar absence of systematic processes at the provincial summits and a lack of follow through between provincial and national summit has been reported on in a previous paper. The draft policy that was available at the start of the national summit was the same draft policy that was available at the start of the provincial summits, suggesting that the inputs at the provincial summits had served the same purpose as those at the national summit. This is reported on in the previous paper.  References to these process issues and issues of follow through between provincial and national summits described in this previous paper have been added to the Context section. | Context: pp. 8-9 |
| 8 | Results/Discussion:  On page 18 the authors state that it is evident that the service user involvement in the national summit appeared tokenistic, but they provide no real justification for this statement aside from the limited number of service user participants. What makes the authors think this? Were the service user knowledge claims treated differently than those of other participants? | As mentioned earlier, it was possible to discern that the majority of speakers giving input in the discussions were academics and/or service providers.  The lack of service user representation and the way in which service user contributions were treated in a ‘tokenistic’ manner have been reported on in a previous paper, where service user representatives who had been involved in organising and/or participating in the summits reported these issues.  Reference to the previous paper in which this limited and tokenistic service user representation is described has been included in the Context section of this paper. | Context: p.9 |
